# Drug-Induced Liver Injury Network Causality Assessment: Criteria and Experience in the United States

**DOI:** 10.3390/ijms17020201

**Published:** 2016-02-04

**Authors:** Paul H. Hayashi

**Affiliations:** University of North Carolina Liver Center, 8011 Burnett-Womack Building, Chapel Hill, NC 27599-8754, USA; paul_hayashi@med.unc.edu; Tel.: +1-919-966-2516; Fax: +1-919-966-4131

**Keywords:** hepatotoxicity, causality, diagnosis, expert opinion

## Abstract

Hepatotoxicity due to drugs, herbal or dietary supplements remains largely a clinical diagnosis based on meticulous history taking and exclusion of other causes of liver injury. In 2004, the U.S. Drug-Induced Liver Injury Network (DILIN) was created under the auspices of the U.S. National Institute of Diabetes and Digestive and Kidney Diseases with the aims of establishing a large registry of cases for clinical, epidemiological and mechanistic study. From inception, the DILIN has used an expert opinion process that incorporates consensus amongst three different DILIN hepatologists assigned to each case. It is the most well-established, well-described and vigorous expert opinion process for DILI to date, and yet it is an imperfect standard. This review will discuss the DILIN expert opinion process, its strengths and weaknesses, psychometric performance and future.

## 1. Introduction

While liver injury from drugs compared to herbal and dietary supplements (HDS) represents different challenges particularly in terms of recognition, dose and agent purity, for the purposes of this review, the term drug-induced liver injury (DILI) will be used to encompass both drug and HDS induced hepatotoxicity. DILI lacks broadly useful or widely accepted objective diagnostic tests, and thus, diagnosis depends largely on clinical acumen. Such means of diagnosis may be more acceptable for common diseases that avail themselves to widely available clinical experience and honing of diagnostic skills. However, the rarity of DILI limits clinical experience for the general practitioner and even gastroenterologists and hepatologists who do not subspecialize in the field of DILI. This rarity and need for diagnostic expertise makes research especially difficult by hindering accrual of large numbers of bona fide cases.

The U.S. Drug-Induced Liver Injury Network (DILIN), which started in 2004, addresses these challenges by creating a large registry of well characterized and prospectively followed DILI patients upon which diagnostic, epidemiologic and mechanistic studies are done [1]. Enrollment is enhanced by drawing cases from several large U.S. referral centers, and diagnostic vetting of cases occurs by a consensus opinion of hepatologists with experience and expertise in the field of DILI. The DILIN protocol for causality assessment is quite specific in its steps and processes. Perhaps its greatest strength and uniqueness is its consensus of opinions by several DILIN hepatologists, so called “experts” in the field, who can weigh in on each and every case. While not a perfect gold standard for diagnosis, it remains arguably the best standard currently available. This review will cover the DILIN expert opinion causality assessment process, its strengths, pitfalls and performance. The protocol has been detailed previously [2]. Therefore, the intent here is to enhance prior descriptions with added information and nuances wherever possible, and to describe what is known about the protocol’s psychometric performance and future.

## 2. The Drug-Induced Liver Injury (DILI) Expert Opinion Process

**Figure 1:**
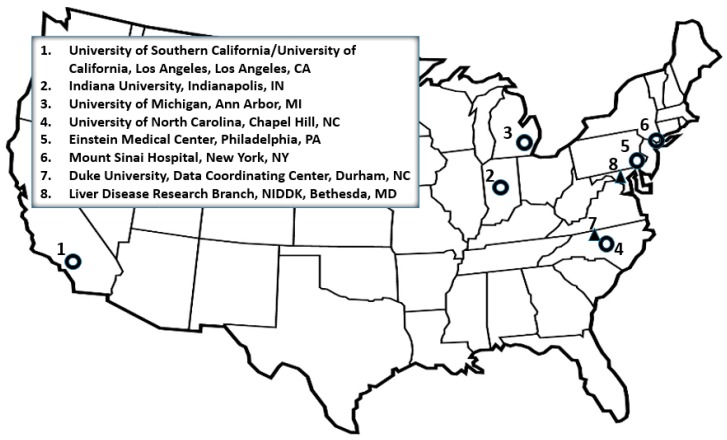
Drug-Induced Liver Injury Network (DILIN) 2015. NIDDK = National Institute of Diabetes and Digestive and Kidney Diseases.

Currently, cases are enrolled from six sites (Figure 1) across the continental U.S. The Los Angeles and North Carolina sites include two large academic centers each, and several sites have outlying satellite clinics expanding their enrollment catchments well beyond their immediate metropolitan areas. All sites are large referral centers for liver disease and transplantation. Each site must go through an entry and then renewal process every five years. The application for entry and renewal is extensive and highly competitive. While there have been site changes over the 12 years, three of the current six sites have remained since inception, and two have been enrolling for more than seven years. Each site maintains institutional review board (IRB) approval at their respective centers. The data coordinating center as well as direct support and oversight by the U.S. National Institute of Diabetes and Digestive and Kidney Diseases (NIDDK) have remained the same since 2004.

In order to enrich the registry with clinically relevant cases and enhance diagnostic accuracy, certain liver biochemistry abnormality thresholds must be met for patients to be enrolled: (a) serum ALT or AST greater than five times the upper limit of normal (ULN) or prior baseline on two consecutive examinations; (b) serum alkaline phosphatase (ALP) greater than two times ULN (c) bilirubin greater than 2.5 mg/dL with any elevation in liver enzymes or (d) INR greater than 1.5 with any elevation in liver enzymes. Patients must be more than two years old and enroll within six months of DILI onset which is defined as the first set of qualifying labs values as described above (List 1).

List 1. Drug-Induced Liver Injury Network inclusion and exclusion criteria
Inclusion Criteria
  - Reasonable suspicion of DILI
  - Age > 2 years
  - Enrolled within 6 months of DILI onset
  - One or more of the following:
    ○ ALT and/or AST > 5 × ULN or prior baseline on 2 consecutive blood tests
    ○ Alkaline phosphatase > 2 × ULN or prior baseline on 2 consecutive blood tests
    ○ Total bilirubin > 2.5 mg/dL and an elevated ALT, AST or alkaline phosphatase
    ○ INR > 1.5 and an elevated ALT, AST or alkaline phosphatase
Exclusion Criteria
  - Acetaminophen hepatotoxicity
  - Primary biliary cirrhosis, autoimmune hepatitis, primary sclerosing cholangitis, chronic biliary tract disease
  - Liver or bone marrow transplant prior to DILI

DILI = Drug or herbal/dietary supplement induced liver injury; ALT = serum alanine aminotransferase; AST = serum aspartate aminotransferase; ULN = upper limit of normal; INR = international normalized ratio.

Important exclusion criteria include acetaminophen induced liver injury, certain chronic liver diseases, and prior bone marrow or liver transplant (Table 1). Chronic hepatitis C and B patients may be enrolled. Patients with HIV infection may also be enrolled.

**Figure 2:**
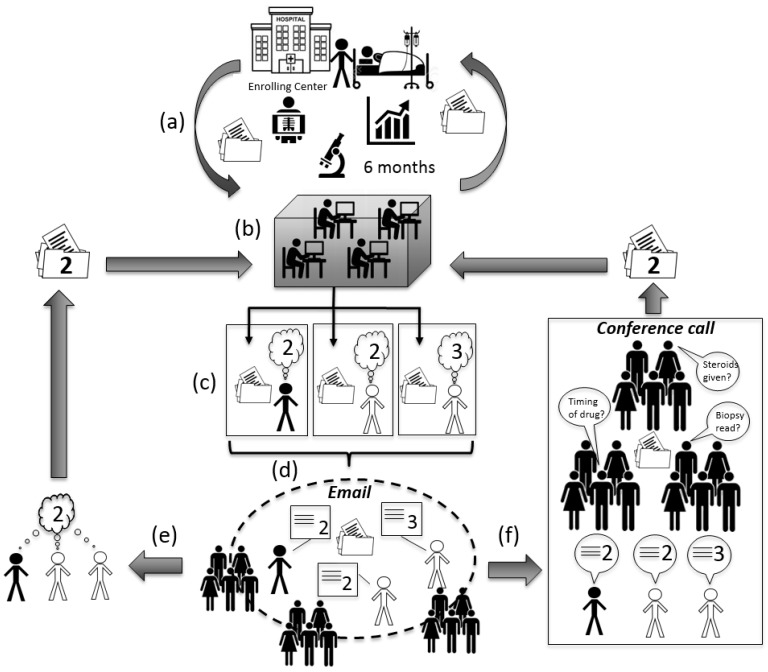
Drug-Induced Liver Injury Network (DILIN) consensus causality assessment. (**a**) Six months of clinical information are collected at the enrolling center and communicated to the data coordination center. Clinical narrative created and approved by enrolling center; (**b**) Narrative and data forms created by the coordinating center; (**c**) Finalized narrative and data forms uploaded to a secure website for the enrolling investigator and two other DILIN hepatologists, chosen at random, to independently review. Reviewers are given two to three weeks to render a causality score on the website. In this example, two reviewers give the case a “2”, but one reviewer renders a “3”; (**d**) Scores are released to the group and email communication between the three reviewers occurs over four days to resolve differences in scoring. All DILIN hepatologists have the opportunity to comment on the case at this stage; (**e**) If consensus amongst the three reviewers is obtained by email, then finalized score is sent back to the data coordinating center to be logged; (**f**) If consensus is not obtained via email, then the case is formally presented and discussed with all DILIN hepatologists by conference call. All attendees have the opportunity to ask questions, make comments and give opinions. If agreement amongst the three reviewers is still not reached, then consensus is obtained by majority vote (one vote per center). Finalized score sent back to the data coordinating center to be logged.

Each case is assessed by one of the DILIN hepatologists at each site for appropriateness of enrollment and reasonable suspicion of DILI. After informed consent, several whole blood, serum and urine samples and imaging studies are obtained. These samples and images are used to look for competing diagnoses (e.g., choledocholithiasis, acute viral hepatitis) and held for future research. Test results already obtained prior to enrollment may be used and not duplicated at the discretion of the on-site DILIN hepatologist. The necessary diagnostics to rule out other competing cause for liver injury have been delineated elsewhere [2,3]. Patients are then followed by the DILIN hepatologists often in conjunction with the referring provider(s) for six months, under routine clinical care (Figure 2). All clinical data for the six months of follow-up are captured and collated in a de-identified research file for each case. Patients are seen again at six months (+/− a 30 day window) specifically for DILIN follow-up, re-evaluation by the on-site DILIN hepatologist and repeat whole blood and serum sampling. Liver biopsy is not required, but every effort is made to obtain liver tissue blocks and slides of any biopsy obtained during the course of routine care. Blocks and slides are sent to a single DILIN liver histopathologist, for over-read and analysis. However, his reading is not a part of the causality assessment at present. Instead, the local reads of the biopsy, either from the referring center and/or the enrolling center, are taken into consideration.

Once the six-month visit is completed, the process of causality assessment begins (Figure 2). The enrolling site prepares a clinical narrative. The narrative identifies up to three culpable agents and summarizes the case, including patient history, agent(s) exposure dates, and reasoning for suspecting DILI. Such narratives are typically one to three pages of single space typing placed on a standard form. In addition, a standardized form including tables of summarized clinical, laboratory, histologic and radiographic data is generated off a central database maintained by the data coordinating center. A final table records liver biochemistries (ALT, AST, ALP, and bilirubin) and INR, over time allowing assessment of DILI onset, course and resolution. This data form is typically five to eight pages long. Once the narrative and data entry are complete, the narrative and data are sent back to the enrolling center for one last review before being approved for causality assessment (Figure 2).

Each case is randomly assigned to two of the DILIN hepatologists for independent review. The third reviewer is always from the enrolling center and, therefore, typically has seen the patient personally. No two reviewers may be from the same DILIN site. The reviewers have two to three weeks to independently score the case as a whole, and each implicated agent, as 1 = definite (>95% likelihood), 2 = highly likely (75%–95% likelihood), 3 = probable (50%–74% likelihood), 4 = possible (25%–49%) and 5 = unlikely (<25%) (Table 1). All assessments are entered on-line at a secure DILIN website. Four days prior to a monthly Causality Committee teleconference, all reviewer identities and their scores are sent to all DILIN hepatologists by email for review. Cases are discussed by email with input from any of the DILIN hepatologists, even those not assigned to a particular case (Figure 2). If consensus is not obtained by email, then the case is discussed on the teleconference that includes all DILIN hepatologists (typically 15–25 hepatologists per call). This monthly telephone conference includes a shared computer screen to display each case’s narrative and clinical data. The case is presented by the reviewer who enrolled the patient. If the teleconference discussion does not produce consensus amongst the three reviewers, then a roll call vote is obtained from the Committee with one vote being cast per center. Majority prevails.

**Table 1:** Drug-Induced Liver Injury Network scoring categories.

Causality Score	Likelihood	Description
1 (Definite)	>95%	Beyond any reasonable doubt
2 (Highly likely)	75%–95%	Clear and convincing data, but not definite
3 (Probable)	50%–74%	Majority of data supports causal relationship
4 (Possible)	25%–49%	Majority of data suggests no causal relationship but possibility remains
5 (Unlikely)	<25%	Causal relationship very unlikely
6 (Insufficient data)	ND	Missing key data

ND = not determinable.

At any point in the assessment process, any of the DILIN investigators may flag a case as being incomplete or request that the case be “recycled” back to the enrolling center for clarification or additional information. Such flagged cases are then postponed to a later round of cases and conference call. Even after final causality assessment is assigned, cases can be reopened for full repeat assessment by three reviewers, if a DILIN investigator feels new information has come to light or any other issues warrant a relook. A patient’s subsequent long-term course may be pertinent to the prior scoring (e.g., later re-challenge, subsequent diagnosis of autoimmune hepatitis) warranting a change. Also, advances in DILI research may require reassessment, as was the case when data on hepatitis E in DILI were published [4,5]. Retrospective testing for HEV of stored serum was done and led to a downgrading of DILI likelihood in a small number of cases. Such ongoing flexibility in the DILIN is crucial to maintaining the veracity of the registry.

## 3. Psychometric Performance

Without a true gold standard for DILI diagnosis, validity cannot be assessed for any current diagnostic method. Only comparisons between methods can be done, and such comparisons have been published including one involving the DILIN expert opinion process [6,7,8]. However, reliability can be assessed without a gold standard and is equally essential. Reliability of the DILIN causality method has been examined for both inter-rater and test-retest reliabilities.

Inter-rater variability, prior to consensus, was evaluated in 250 DILIN cases where a single agent was identified [6]. Weighted κ for complete agreement amongst all three reviewers was low at 0.23 and comparable to other studies comparing individual expert opinion on adverse drug reactions in general (κ scores of 0.05 and 0.2) [9,10]. However, the mean absolute difference in categorical score between reviewers was low at 1.1. In other words, on average, reviewers disagreed by one category score only. In 27% of cases, all three reviewers independently agreed without need for consensus discussion. In 43% of cases, two reviewers agreed and the third differed by just one score category. More importantly, the independent individual assessments are not the end-product of the DILIN Causality Assessment. The problem of inter-rater variability amongst experts was anticipated. Therefore, the consensus process was built into the DILIN protocol to mitigate this variability and produce a committee-derived assessment as its final output.

Because the consensus score is the final output, the test-retest reliability for the overall DILIN consensus assessment is a better measure to consider. Prior to April 2009, the DILIN assessed and adjudicated cases as soon as they enrolled. Thereafter, cases were adjudicated six months later to allow follow-up data to inform the assessment process. One hundred cases were randomly chosen for retesting, but stratified 1:1 before and after April 2009 [11]. Cases were completely re-evaluated by three different DILIN reviewers. Case narratives and data files were re-issued to the new reviewers without the previous scores. The median time between reviews was 938 days (range 140–2352). The DILIN adjudicates 10–30 cases per month, so it is unlikely any of the reviewers would recall the previous scores for any particular case after so many days. Weighted κ for agreement was 0.60 for all 100 cases. For the 51 cases enrolled after April 2009, the weighted κ was higher at 0.67. The difference between a score of 1 (definite, >95% likelihood) and 2 (highly likely, 75%–95%) is clinically much less relevant than the differences between the other three scores (Table 1). Therefore, the two highest likelihood scores were combined for a sub-analysis. Using such a four-category scale (1–2, 3, 4 or 5), the κ rose to 0.73 after April 2009, with 73% coming to the same consensus score on retest, and 25% differing by one category. Only 2% (one case) differed by more than two categories on retest. Still, 12% of cases since April 2009 were particularly challenging and had a change in score that crossed the 50% likelihood of DILI line, typically being reassessed as less likely to be DILI.

## 4. Strengths of the DILIN Causality Assessment

Idiosyncratic DILI covers a broad range of unique and even peculiar presentations of hepatotoxicity. A one-size fits all diagnostic algorithm is unlikely to cover the gamut of presentations as well as a group of experts. Medications with very long latencies such as nitrofurantoin or minocycline will typically score lower on any algorithm aimed at diagnosing the majority of DILI where latencies are usually much shorter. On the other hand, a group of experts in the field will see these cases as definite, even classic, cases. Clear-cut cases of isoniazid acute liver failure or carbamazepine DILI with DRESS, where the liver enzymes are not allowed to decline due to intervening events (e.g., liver transplant, sepsis), will be rated more highly by an expert than algorithms that require decline in liver enzymes to get a high score [7,12].

Also, using the opinion of a group of hepatologists with a research interest in DILI is arguably the best way to keep a DILI assessment process current. Such reviewers are the most likely to be abreast of recent publications making the process self-updating. This can be crucial when suspected agents are relatively new to the market and reported cases of hepatotoxicity quite recent and scant.

The DILIN protocols’ use of consensus probably offers its most important strength. As already discussed, the DILIN consensus process does have reasonable reliability on retest, something no other DILI diagnostic process or tool has demonstrated. Validity matters little if a process cannot be reliably replicated. Consensus prevents one expert opinion that may be an outlier to control the assessment of a case. Indeed, each case is open to comment and input from all DILIN hepatologists at any time after the initial independent reviewer scores are in. Strong opinions or biases of a single expert can be attenuated by others. Lengthy and lively conversations often occur in the DILIN consensus process, as reviewers point out overlooked data, weakness in reasoning, or data from new publications. Certain DILIN hepatologists have a known expertise in a particular type of DILI and their opinion is routinely sought during discussions. In these ways, the collective wisdom and experience of the whole DILIN group are brought to bear on each case.

## 5. Weaknesses and Limitations of the DILIN Causality Assessment

The DILIN process is cumbersome, time-consuming and costly. Since 2009, final preparation of DILIN case documents for causality assessment does not even begin until six months after a case enrolls. Preparation and approval of documents for distribution to the three reviewers can take several weeks to months beyond that. The logistics of the consensus process requires administrative, organizational, and technologic expertise. A large group of ancillary staff are needed at the data coordinating center and at each site. Reliance on information technology is heavy. Case documents and assignment to reviewers are posted on a secure DILIN website for all members to access under password protection. Monthly meetings occur in a teleconference fashion with a shared viewing screen. Needless to say, this process is impossible to reproduce without internet expertise and is completely inaccessible to the clinician needing a causality assessment in real time. Even for research purposes, the process is slow.

There is no accepted definition for an expert in DILI. The DILIN consists of highly experienced hepatologists, all with a keen interest in DILI, but no further criteria are delineated. However, each academic center must competitively apply and renew every five years for inclusion in the DILIN. Experience and publication records of the principle investigators (reviewers) at each site are a significant part of the application and renewal evaluations.

Consensus does not necessarily equal truth. It is always possible that the minority or single voice is closer to the truth. Also, consensus can fall victim to too much compromise, since cases are not allowed to be left unresolved. In other words, there is no “hung jury” option. Consensus for each case must be reached. After lengthy discussions, a reviewer may capitulate in the name of compromise, knowing their voice is in the minority and will lose on a vote. When reviewers do hold steadfastly to their opinion, then outcome is by majority vote. There is little science behind such a democratic process. No doubt there are times when those of the minority opinion begrudgingly abide by the final assessment, believing that a wrong decision was produced by the process. Such cases typically fall in the 3 and 4 score categories, where the likelihood of DILI hangs in the balance around the 50% likelihood. In fact, as mentioned, about 12% of DILIN cases cross this line on re-test, and represent some of the most challenging cases to adjudicate. So, expert opinion, even when by consensus, is still an opinion—one formed by a large group of experts in the field, but a subjective opinion, nonetheless. It is the best standard we have for the time being, but it is an imperfect standard.

## 6. Improvements and Future Directions

Mechanistic studies are making progress identifying markers of DILI and DILI risk. These studies hold the greatest promise for true “gold standards” for DILI diagnosis. Many studies have found associations between DILI and polymorphisms in genes that regulate drug metabolism including the cytochrome P450s, and various transferases and transporters [13,14,15,16,17]. There is also great interest in the immunologic bases of DILI based on associations found with several HLA genotypes, both class I and II [18,19,20,21,22,23]. However, so far, none have found their way into causality assessment or clinical practice as standards for diagnosis.

Therefore, the DILIN expert opinion process or similar will likely remain as an important research tool for the foreseeable future [14,18,19]. Improving the DILIN process is also a continuing mandate. Process changes to improve presentation, communication and assimilation of case data have occurred over the years (e.g., incorporation of better computer and information technology). The largest change in the process came in 2009 as mentioned earlier, but other smaller ways to improve the process are continually sought.

For example, how and whether to incorporate the DILIN expert reading of liver biopsies is being considered. Liver biopsy findings from the DILIN have been recently published [24], but incorporating such data into causality assessment is a challenge. Biopsies, when obtained, are read centrally by the DILIN histopathologist. Incorporating such expert readings into causality would slow the process further as slides are retrieved, re-stained and methodically read and scored both blinded and unblinded to clinical data. Doing so would also take the process further from “real world” practice where an expert liver histopathologist is not typically at hand. Lastly, the DILIN has no consensus process for the histologic reading, having just one pathology expert, as opposed to three reviewers for all other case data. Nevertheless, incorporating the DILIN histopathologist’s reading is desirable, if it can be done.

Herbal and dietary supplements (HDS) present unique challenges to hepatotoxicity assessment and has risen in incidence within the DILIN [25,26]. Precise details on agent contents are often unclear or unavailable, and mixtures of compounds are more common compared to medications. Literature on liver injury from HDS is less robust compared to pharmaceuticals. Such differences present challenges for the DILIN causality assessment. For example, the current process restricts the naming of culpable agents to 3. This limitation can be difficult when several HDS products each with little in the way of published literature are being considered. Adulteration, contamination and inconsistencies in HDS products batch-to-batch can make determination of true exposure and latency for a particular hepatotoxic ingredient unclear [27,28,29]. These issues may hinder the performance of the DILIN causality assessment process for HDS products. Efforts to address these issues by a DILIN HDS subcommittee are ongoing.

External validity would be an important performance metric to address. The DILIN investigators have become experienced in the consensus process. Their skills continue to evolve. The group understands the protocol well and the sorts of cases that are worthy of higher likelihood scores. The group also took a systematic look at those cases scoring a 5 (unlikely DILI) to see what characteristics lead to mistaken DILI [30]. Undoubtedly, this type of feedback affects the DILIN reviewers as they assess future cases. Also, the DILIN has always been restricted to U.S. centers. After more than 10 years in use, it is unknown whether the process can be quickly reproduced with a different set of investigators, country or patient population. Validating the process with a completely different group of experts would be worthwhile, but labor intensive and expensive.

Testing validity of the DILIN causality assessment against future objective tests will eventually come. Only then will we know the true metal of the process and the strength of the registry. Smaller ancillary studies looking at specific biomarkers are ongoing, but for the most part, these test the new biomarker *against* the DILIN assessments as a standard and not the other way around.

## 7. Conclusions

The diagnosis of DILI remains problematic. Efforts to develop better diagnostic tools, including algorithmic score cards, statistical modeling and use of blood test markers, are underway. However, until these endeavors mature, the DILIN causality assessment process, despite its shortcomings, remains an important method in the field and a reasonable diagnostic standard. Certainly, it has created a registry that can serve as a platform upon which to build better diagnostics using statistical modeling and/or biomarkers. Indeed, a primary goal of the DILIN expert opinion process was always to make itself obsolete, in favor of something easier, more accessible and with robust validity.
